# Dynamics of Hepatitis B infection prevention practices among pregnant women attending antenatal care at Lubaga Hospital Kampala, Uganda using the constructs of information-motivation-behavioural skills model

**DOI:** 10.1186/s12889-022-14723-3

**Published:** 2022-12-01

**Authors:** Ismail Bamidele Afolabi, Abdulmujeeb Babatunde Aremu, Lawal Abdurraheem Maidoki, Nnodimele Onuigbo Atulomah

**Affiliations:** 1 Faculty of Science and Technology, Department of Public Health, Cavendish University, Kampala, Uganda; 2 Faculty of Health Sciences, Department of Human Anatomy, Islamic University in Uganda, Kampala Campus, Kampala, Uganda

**Keywords:** HBV infection, Knowledge, Perception, Behavioural skills, Prevention practices, Pregnant women

## Abstract

**Background:**

Hepatitis B virus (HBV) infection is considered a significant global public health challenge with infectivity as well as estimated potential for transmission more than 50 to 100 times that of HIV. Over time, numerous empirical studies have shown that majority of HBV-related yearly global deaths are secondary to carcinoma of the liver. It is also known that HBV infected Women have the potential to transmit the infection vertically to their infants during pregnancy. This accounts for the WHO reported 3.16% prevalence among children less than 5 years of age in Uganda. This study assessed the predictors of HBV infection prevention practices among eligible consenting pregnant women using Lubaga health facility for antenatal care (ANC).

**Methods:**

A cross-sectional descriptive study employing quantitative data collection based on the constructs of IMB model was used to capture data on the study variables among 385 randomly selected eligible pregnant women attending antenatal care at Lubaga hospital between September 2020 and October 2020. Data derived from the quantitative instrument was analysed by data reduction and transformation to summaries of descriptive statistics using (SPSS version 26) and regression analysis was performed to establish characteristics of the association between the variables with significance level set as (*p* < 0.05). Chi-square goodness-of-fit test was employed for significant differences in the proportion of dichotomous responses.

**Results:**

The findings showed that more than half of the respondents (59%) were between the ages of 18 and 28 and majority of them (42.3%) had secondary education. Furthermore, an average but inadequate knowledge ($\overline{X }=$ 5.97 ± 6.61; B = 0.57; *p* < .001), positive perception ($\overline{X }=$ 17.10 ± 18.31; B = 0.97; *p* = .014) and good behavioural skills ($\overline{X }=$ 12.39 ± 13.37; B = 0.56; *p* < .001) for adopting prevention practices all statistically predicted the averagely acceptable level of prevention practices ($\overline{X }=$ 15.03 ± 16.20) among the study respondents as measured on rating scales of 12, 33, 21 and 30 respectively.

**Conclusion and recommendation:**

There were observed gaps in their knowledge about some basic features of the infection like transmission and risk factors as well as some misperceptions about vaccination despite the relatively average score level for both, which is likely to influence their prevention behaviours and predispose them to the risk of the infection if actions are not taken. Therefore, personalized health education is needed during antenatal visits and subsequent health campaign in order to inform better prevention practices among this vulnerable population group.

**Supplementary Information:**

The online version contains supplementary material available at 10.1186/s12889-022-14723-3.

## Background

Hepatitis B virus infection is considered a serious global public health challenge with infectivity rate, as well as potential for transmission being fifty to hundred times more than that of HIV [1]. Previous report from Global burden of disease (GBD) show that an estimated 68,600 people died as a result of complication from HBV infection only in 2013. Numerous reports have also shown that 300,000 to 786,000 HBV-related deaths are secondary to carcinoma of the liver every year globally [2] which makes it ranked globally as the tenth-leading cause of mortality [3, 4]. It is also known that HBV infected women have the potential of passing on the disease vertically to their infants [5, 6].

It is estimated that, globally, prevalence of carriers of the virus is approximately 240 million individuals [7]. The inhabitants of the Western Pacific region that have more than 95 million chronically infected people are the largest population with chronic HBV infection, succeeded by more than 75 million individuals that are chronically infected in the African region [8].

Specifically, a country like Uganda has documented a much increased magnitude of hepatitis B virus infection than any other neighbouring regions within East Africa. It has remarkable regional differences in the burden of the infection with the national figures posing a mean value of 10% [5, 9] in comparison with 6% and 6.9% in counties like Tanzania and Ethiopia respectively [6, 10] and also boasting regional variations of 4.6%, 4.4% and 3.8% for Northern region, North East and in West Nile respectively leaving a lower rate in the remaining parts of the country ranging from East-Central region to South-West region with a proportion of 2.7% and 0.8% respectively [11].

In Uganda, Hepatitis B infection is extremely endemic, with infectivity occurring at adulthood as well as childhood [5]. Over 1.4 million chronically infected adults are said to be estimated and few regions reported to be affected out of proportion [5]. Among pregnant women aged 13 to 43 years whether married or cohabiting, hepatitis B infection prevalence was recently reported to be 11.8% in a previously conducted study by Bayo et al., [12] in Northern Uganda and the highest positivity rate for HBsAg was observed as 20% in women aged 20 years or less while women above the age group displayed 8.7%. Due to insufficient information on pregnant women’s health belief, it remains unclear, whether HBV infection information-adequacy in this population that contributes greatly to the vertical transmission of the infection is accurate or the differences in their knowledge gaps reflect the ones demonstrated in the disease magnitude.

The behavioural theory (IMB model) postulated in early twentieth century by Fisher affirms that information related to health or information-adequacy about health conditions, motivation, as well as behavioural skills are the leading predictors of performance of health behaviours and adoption [13]. Similarly, the health belief model of Becker, [14] asserts that performance or not engaging in health-promoting behaviours is explained by an individual’s beliefs about health-related events, outcome expectancy perceived, self-efficacy and perception of barriers to action.

More so, the documentation regarding the degree of information-adequacy on HBV infection, burden, transmission, prevention as well as perception and behavioural skills towards HBV prevention practices among expectant mothers within central region of Uganda is limited. The lone research on knowledge and awareness was conducted in Kiswa and Kasangati health centres III and IV respectively by Nankya-Mutyoba et al., [15] and within Sub-Saharan Africa, the few works to have evaluated awareness and the disease knowledge among pregnant women were mostly carried out within Western Africa [16, 17] and Central Africa [18, 19]. These regions have variations in cultures and infection magnitude compared with that of East Africa and Uganda and in view of this present gap in hepatitis B virus infection research, there would be a challenge to health facility intensification for hepatitis B virus infection elimination through health education and immunization.

Hepatitis B infection prevention practice as a health-related behaviour can be affected by the level of adequacy of information regarding the disease, motivation towards the disease prevention as well as the behavioural skills initiated for the disease prevention in accordance with the information-motivation-behavioural skills model [13].

Therefore, in a general health facility in central Uganda, assessing the awareness and knowledge of pregnant women, motivational perception and behavioural skills towards HBV infection prevention practices would provide accurate and significant information needed for controlling mother to child transmission of this infection. This is because, if pregnant women are accurately and adequately informed about HBV infection, its transmission and prevention via immunization, they will tend to use the health care facility more, to request and get screened, ensuring protection of their unborn babies, as numerous reports from comparable diseases of infectious origin have demonstrated the same [20, 21]. Hence, the study sought to assess knowledge of HBV infection, perception, and behavioural skills as predictors of HBV infection prevention practices among pregnant women in a general health facility in Central Uganda.

## Methodology

### Research design

The study is a cross sectional descriptive study that employed quantitative data approach to capture data on the study variables among the pregnant women using Lubaga health facility for antenatal care between the period of September 2020 and October 2020, since antenatal clinic settings would be appropriate to collect pertinent information related to hepatitis B virus infectivity.

### Study location

This study took place at Lubaga hospital, which was formerly known as Uganda Martyrs hospital. It is a private not-for-profit health facility owned by Kampala Roman Catholic Archdiocese. Over the years, the hospital, which is relatively a high-volume 237 bed-capacity facility has established a name as a highly as an affordable health care provider. Consequently, it is seen as having persistency allures and a center that manages treatment of individuals from both the low and middle socio-economic status. Millions of people have been reported to have benefited from its health care services during its long history of existence. This hospital is also known to have built a very strong anecdotal reputation for treatment/ management of liver diseases ever since the 1970s where anybody with liver problems in Uganda was often referred to. Antenatal clinics operate from Mondays through Saturdays in the Public Health Department of the hospital that has more than 400 antenatal clinic attendees per week.

### Inclusion and exclusion criteria

Eligible participants were expectant mothers of not less than 18 years, who were on routine antenatal care in Lubaga hospital and who gave their written consents to be part of the procedures of the study. Participants who were severely indisposed (i.e. those who were extremely exhausted) to embark on the study-specific procedures were excluded.

### Sample size and sample techniques

The study involved a total of 385 respondents from Lubaga Hospital, calculated employing Kish Leslie (1965) formula on the basis of the previously reported prevalence of 47% awareness of HBV infection by Nankya-Mutyoba et al., [15], 0.05 precision level and 5% (**α**) or type 1 error.

$$N=\frac{Z{\mathrm{\alpha }}^{2}pq}{{d}^{2}}$$

where N denotes the estimate of the sample size for the pregnant women

$$P=reported\;prevalence\;of\;HBV\;awareness\;(47\%)$$

$$Z\alpha=1.96\;value\;for\;the\;confidence\;interval\;at\;95\%$$

$$d=5\%\;error\;of\;maximum\;acceptability$$

The pregnant women were sampled using systematic-sampling approach where every registered 2^nd^ pregnant woman waiting to be attended to was selected to participate in the study following a random start. Recruitment of the participants was based on the daily patronage level and available population at the ANC unit of the Public Health Department. As the expectant mothers arrived to the facility, they were registered before being recruited using the periodic interval. Therefore, approximately 30 expectant mothers were recruited per day from Mondays through Fridays for a period of 3 weeks.

### Variables

#### Independent variables and dependent variable

The independent variables measured include the information on the socio-demographic characteristics, the level of information-adequacy (awareness and knowledge) measured dichotomously on a 13 point reference scale, level of motivation (perception) measured on a rating scale of 33 using Likert scale responses of 4-point: (“very high”, “high”, “low”, “very low”) and the level of behavioural skills towards HBV prevention practices measured on a similar Likert scale of 4-point including responses: (“strongly agree”, “agree”, “disagree” as well as “strongly disagree”). Hence, the behavioural skills were measured on a rating scale of 21.

The dependent variable (main outcome) measured was the self-reported HBV prevention practices among pregnant women using Lubaga health facility for antenatal care measured with 11 questions on a Likert scale responses of 4-point: (“not at all”, “rarely”, “occasionally” and “very often”). Therefore, the practice of HBV prevention was measured on a total reference scale of a 30. All responses to the 11 questions on the infection prevention practices were transformed to rating scale by computation to generate the overall score and summaries of descriptive statistics.

#### Instrument validity and reliability

The validity of the instrument was assured by designing the constructs based on the theoretical framework of health behaviour operationalized in the Information, Motivation, and behavioural skills (IMB) behaviour diagnostic model. Literature review offered confirmation of the appropriateness of the variables in the study and the Cronbach’s internal consistency for items operationalizing the variables was 7.8 for the pilot of the instrument. A test and retest reliability coefficient of 8.4 indicated adequate reliability of the instrument.

#### Data collection procedure

The study was conducted at Lubaga Hospital. Two research assistants specialized in public health or Nursing/ midwifery that had completed NIDA Good Clinical Practice online ethics training course (https://gcp.nidatraining.org/) were recruited to collect data. All research assistants were trained on study specific processes. They were provided with written materials and inclusion and exclusion criteria. The research assistants introduced the aim and obtained oral consent from the eligible pregnant women and then administered the questionnaires to them while also assisting them with any issue arising from completing the questionnaire.

#### Data analysis and test of significance

The collected raw data captured with the questionnaire were entered into version 26 of SPSS where transformation by computation was carried out to generate statistics which are descriptive prior to using the result for further statistics inferentially. Univariate descriptive analysis of socio-demographic, disease-specific knowledge, perception, behavioural skills and prevention practices variables was performed by computing the items under each and every predictor variable to assess the level from their respective mean scores and reported by mean values and percentage mean scores (prevalence). While Analysis of variance was performed to test differences in the level of HBV prevention practices across the demographic characteristics, Chi-square non-parametric (goodness-of-fit) test was employed to test the differences in the proportion of the dichotomous responses of the constructs of information-adequacy.

Linear regression analysis was done to establish the degree of association between the computed variable of prevention practices with the IMB constructs and a 5% cut-off set for level of significance.

#### Ethical issues

Ethical approval to collect the data was granted by Lubaga Hospital following thorough review of the proposal by the hospital ethical review committee (LHREC) to ensure the study is generally satisfactory in terms of safety and protection of the rights of human subjects with a referenced protocol number LHREC/2020/ 07 (Refer to Appendix 1). To participate, prospective participants were duly educated vis-à-vis the aim, benefits and risks involved during the course of the study’s investigation. Subsequently, interested participants gave their written consents.

## Results

### Socio-demographic characteristics

The study captured data from 385 respondents. It was observed that more than half of the participants (59%) were between the age of 18 and 28 and the least age group (2.1%) lies between the age of 40 and above. Majority (272/385 (70.6%)) were from the central region while nearly half of the respondents 163 (42.3%) had secondary education. More than two-thirds (296/385(76.9%)) of them reported to be in monogamous affair while majority (140/385, 36.4%) were also reported to be Catholics with most of them, 158 (41.0%) being self-employed. Upon the assessment of the mean scores on HBV infection prevention practices among the respondents, younger women (14.9), respondents from other regions (14.7), women with non-formal education (14.4), single/divorced women (14.1), and traders (14.4) demonstrated the poorest scores for HBV prevention practices (see Table 1).

**Table 1:** Demographic characteristics of the respondents

Respondents in the study *N* = 385			
Variables	Frequency (N)	Percentage (%)	Mean scores and 95% CI on HBV Prevention Practices
**Age (in years):**			
• 18–28 years	227	59	14.9 (14.10–15.64)
• 29–39 years	150	39	16.6 (15.65–17.47)
• 40 years and above	8	2.1	19.1 (14.51–23.74)
**Region of birth:**			
• Central	272	70.6	16.0 (15.30–16.69)
• Others	97	25.2	14. 7 (13.50–15.84)
• North	16	4.2	14.9 (11.66–18.22)
**Educational attainment:**			
• Secondary	163	42.3	14. 4 (13.45–15.25)
• University	120	31.2	16.7 (15.70–17.65)
• Post-secondary	81	21.0	16.8 (15.56–18.05)
• Primary	19	4.9	15.6 (12.01–19.25)
• Non formal	2	0.5	7.50 (1.15–13.85)
**Marital status:**			
• Monogamy	269	76.9	15.7 (15.00–16.31)
• Single/divorced	56	14.5	14.1 (12.40–15.74)
• Polygamy	33	8.6	17.9 (15.99–19.89)
**Religion:**			
• Catholic	140	36.4	15.8 (14.87–16.96)
• Others	91	23.6	14.5 (13.38–15.74)
• Islam	81	21.0	16.1 (14.86–17.26)
• Protestant	73	19.0	15.9 (14.54–17.21)
**Occupation:**			
• Self-Employed	158	41.0	16.4 (15.51–17.30)
• Housewife	72	18.7	13.1 (11.73–14.52)
• Others	68	17.7	15.0 (13.53–16.41)
• Trader	31	8.1	14.4 (12.47–16.37)
• Teacher	28	7.3	17. 6(15.81–19.33)
• Civil Servant	16	4.2	17.2 (14.69–19.68)
• Health worker	12	3.1	20.3 (16.61–24.06)

### IMB-constructs

#### Information-adequacy (awareness and disease-specific knowledge)

The findings from this study demonstrated that majority 356 (92.5%) had ever heard of hepatitis B as an infection with 333 of them (86.5%) having heard about it before pregnancy. Reported from this study regarding the disease-specific knowledge, majority 216 (56.1%) answered correctly to the item asking if HBV infection is passed from person-to-person via contact with the body fluid of a person infected, more than two-thirds of the respondents (263/385(68.3%)) responded correctly that hepatitis B infection is not caused by taking too much sugar. More than half of them 216 (56.1%) did not know that avoiding multiple sexual partner protects one from getting hepatitis B infection (See Table 2 for full details).

**Table 2:** Findings on information-adequacy (awareness and disease-specific knowledge)

Respondents in this study *N* = 385				
**Statement of consideration**	**Yes (%)**	**No (%)**	**χ**^**2**^	***p*-value**
**Have you ever heard of hepatitis B as an infection**	356 (92.5%)	29 (7.5%)	277.738	< 0.001***
**From where did you hear about it**				
Never heard of HBV	Radio/Television	Churches/Mosques	Others	
29 (7.5%)	169 (43.9%)	3 (0.8%)	184 (47.8%)	
**When did you hear about it**				
Never heard of HBV	Before pregnancy	During pregnancy		
29 (7.5%)	333 (86.5%)	23 (6.0%)		
**HBV infection is transmitted via contact with body fluids of infected persons**	216 (56.1%)	169 (43.9%)	5.738	0.017***
**Hepatitis B infection is caused by taking too much sugar**	122 (31.7%)	263 (68.3%)	51.639	< 0.001***
**Avoiding multiple sexual partners protects one from getting hepatitis B infection**	169 (43.9%)	216 (56.1%)	5.738	0.017***
**Hepatitis B produces fever as an important symptom because the infection does not always show signs and symptoms**	163 (42.3%)	222 (57.7%)	9.042	0.003***
**Untreated Hepatitis B infection can destroy the Liver**	186 (48.3%)	199 (51.7%)	0.439	0.508
***A pregnant woman needs to be protected from having Hepatitis B infection because:***				
** She may pass the infection if she is infected to the baby through drinking water**	205 (53.2%)	180 (46.8%)	1.623	0.203
** Her risk of transmitting the infection to the baby during delivery and breast feeding is high**	241 (62.6%)	144 (37.4%)	24.439	< 0.001***
** She may likely not be able to carry the pregnancy to term**	294 (76.4%)	91 (23.6%)	107.036	< 0.001***
** The baby may die in the womb before delivery because of the infection by the mother**	285 (74.0%)	100 (26.0%)	88.896	< 0.001***
***Hepatitis B transmission is more common among people noted for:***				
** Sharing and reusing needles and injections**	202 (52.5%)	183 (47.5%)	0.938	0.333
** Making tattoos marks on their skin or piercing their nose or ears**	125 (32.5%)	260 (67.5%)	47.338	< 0.001***
** Vaccination during birth can protect the baby from hepatitis B infection and risk of Liver cancer**	297 (77.1%)	88 (22.9%)	113.457	< 0.001***
** To complete the HBV vaccine series that offer 10 years or lifetime protection 1 or 2 doses are generally required**	197 (51.2%)	188 (48.8%)	0.210	0.646

Table 2 above also showed a chi-square-non-parametric test to demonstrate the significant differences in the proportion of the responses of yes and no for the constructs information- adequacy

*** significant at *p* ≤ 0.005

#### Motivation (perception)

The findings presented here display the perception towards hepatitis B prevention practices where there is a very high likelihood that less than half of the total respondents 185 (48.1%) will go for HBV screening and vaccination because a health worker recommended them, and the chance is high for roughly 4 out of every 10 respondents 157 (40.8%) practicing safer sexual behaviour because they were educated that it prevents HBV infection. The probability of nearly one-third of them: 116/385 (30.1%) not going for HBV test because their clinic does not have it as a routine is high and for just 108 (28.1%) that constitutes the majority, there is a low likelihood of not getting themselves and their infants vaccinated because the antenatal clinic is far from their places of residence. Similarly, relatively a third (35.3%) perceived their chance of getting infected with hepatitis B in their lifetime to be very low while the perception of nearly half of them 177 (46%) not likely to get liver cancer in their lifetime if infected with HBV infection is very high (refer to Table 3 for details)**.**

**Table 3:** Findings on the motivation (Perception)

Respondents in this study *N* = 385								
**Statements for Consideration**	**Very High**		**High**		**Low**		**Very Low**	
	**N**	**%**	**N**	**%**	**N**	**%**	**N**	**%**
**• What is the likelihood that you will go for HBV screening and vaccination because a health worker recommended you to?**	185	48.1	136	35.3	34	8.8	30	7.8
**• What is the likelihood that you will practice safer sexual behavior because you were educated that it will prevent HBV infection?**	156	40.5	157	40.8	56	14.5	16	4.2
**• You are not likely to go for HBV test because it is not a routine in the clinic you go**	116	30.1	70	18.2	115	29.9	84	21.8
**• It is likely that you will not get yourself and your infant vaccinated because the antenatal clinic is far from where you live**	97	25.2	76	19.7	108	28.1	104	27.0
**• You are likely to get hepatitis B infection in your lifetime**	106	27.5	42	10.9	101	26.2	136	35.3
**• Likelihood of not getting carcinoma of liver in your lifetime, if infected with HBV infection**	177	46.0	75	19.5	60	15.6	73	19.0
**• Likelihood compared to a woman of your age group of getting cancer of the liver in your lifetime**	102	26.5	28	7.3	132	34.3	123	31.9
**• Likelihood in your lifetime compared to your spouse of not getting liver carcinoma**	163	42.3	93	24.2	65	16.9	64	16.6
**• Likelihood of your under 5 child getting HBV infection if you are infected**	113	29.4	55	14.3	132	34.3	85	22.1
**• How unlikely is it that your spouse will get infected with HBV infection in his lifetime**	168	43.6	109	28.3	54	14.0	54	14.0
**• Likelihood of taking your child for HBV infection vaccination as part of scheduled routine immunization for infants**	133	34.5	110	28.6	68	17.7	74	19.2
**• Likelihood of getting screened for HBV infection over the next 365 days?**	77	20.0	80	20.8	98	25.5	130	33.8

#### Behavioural skills

The findings on the behavioural skills of the pregnant women towards HBV infection prevention practices are displayed in Table 4. In terms of their certainty of HBV infection severity, relatively half of them 216 (56.1%) strongly agreed that HBV infection is a serious infection that can be fatal and should they develop cancer of the liver it will be critical more than any other complications that endangers career and deteriorates financial status. Regarding the outcome expectancy, almost half of the participants, that is, a total of 188 (48.8%) agreed that they were confident that getting screened for hepatitis B infection will help them discover and treat the infection quickly before it leads to liver carcinoma. Slightly one-thirds of them 136 (35.3%) also strongly agreed that they confidently believed that vaccinating their child for hepatitis B infection alleviate their worries about the baby developing carcinoma of the liver during adulthood (refer to Table 4 for full details).

**Table 4:** Findings on the behavioural skills

Respondents in this study *N* = 385								
	**Strongly Agree**		**Agree**		**Disagree**		**Strongly Disagree**	
**Statements for Consideration**	**N**	**%**	**N**	**%**	**N**	**%**	**N**	**%**
**• Confidence on severity of HBV infection and effect of Liver Cancer on career and financial status**	216	56.1	75	19.5	69	17.9	25	6.5
**• Confidence that early screening for HBV helps in treating and avoiding liver cancer**	174	45.2	188	48.8	19	4.9	4	1.0
**• Confidence that there will be no worries for me about my babies developing liver cancer at adulthood should I vaccinate him/her**	136	35.3	91	23.6	57	14.8	101	26.2
**• Confidence that liver infection symptoms is not a necessity for HBV test and vaccination**	124	32.2	73	19.0	59	15.3	129	33.5
**• Confidence that miscarriage will result from getting vaccinated for HBV during pregnancy**	154	40.0	66	17.1	75	19.5	90	23.4
**• Confidence of taking my child for all required vaccinations regardless of how far the immunization center is from my residence**	131	34.0	130	33.8	69	17.9	55	14.3
**• Certainty of going for HBV screening irrespective of the payment it requires**	123	31.9	159	41.3	54	14.0	49	12.7

### Outcome variable

#### Prevention practices of HBV infection

**Fig. 1:**
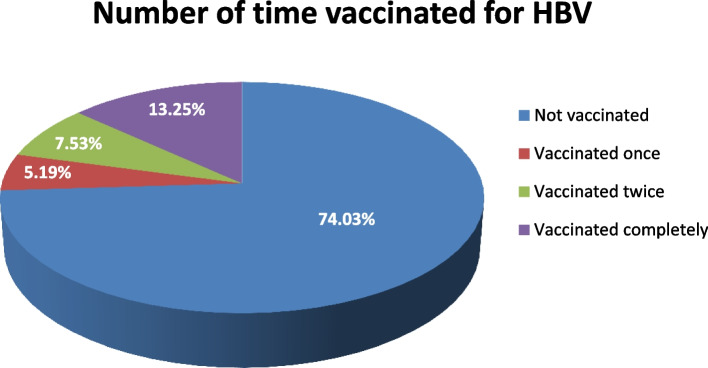
Findings on the number of time vaccinated against HBV infection

The frequency of the responses on HBV infection prevention practices as measured in this study showed that only 4 out of every 10 respondents 152 (39.5%) try to avoid unprotected sex with the use of barrier protective measure like condoms very often. Most of them 263 (68.3%) also often practice safer sex and avoid having more than one sexual partners. While 7 out of every 10 respondents 271 (70.4%) do avoid having tattoos on their body, relatively one-thirds of them 131 (34.0%) reported not to take precaution, hence frequently piercing their ear and nose perhaps because they do not recognize them as risk factors of HBV infection (see Table 5 for full details). In terms of vaccine uptake, nearly three-quarters of the respondents 285 (74.0%) had not been vaccinated for HBV before (refer to Fig. 1).

**Table 5:** Findings on the Hepatitis B prevention practices

Respondents in this study *N* = 385								
	**Not at all**		**Rarely**		**Occasionally**		**Very often**	
**Statements for Consideration**	**N**	**%**	**N**	**%**	**N**	**%**	**N**	**%**
**• I try to avoid unprotected Sex with the use of barrier protective measure like condom**	110	28.6	51	13.2	72	18.7	152	39.5
**• I practice safer sex and avoid having more than one sexual partners**	14	3.6	21	5.5	87	22.6	263	68.3
**• I avoid having tattoos on my body**	28	7.3	22	5.7	64	16.6	271	70.4
**• I do not pierce my ear or nose as a precaution**	131	34.0	74	19.2	57	14.8	123	31.9
**• I avoid casual contact with HBV infected person**	25	6.5	53	13.8	113	29.4	194	50.4
**• I do not share and reuse needle or injection**	109	28.3	90	23.4	42	10.9	144	37.4
**• I avoid sharing food with infected person to prevent HBV infection**	53	13.8	28	7.3	140	36.4	164	42.6
**• I do not remember to wash my hand after contact with body fluid of others**	128	33.2	104	27.0	106	27.5	47	12.2
**• I do not get routinely screen for detection of HBV infection during ante-natal visits**	82	21.3	45	11.7	47	12.2	211	54.8
**• I complete recommended vaccinations for my child against HBV infection as part of routine infant recommendation**	280	72.7	25	6.5	19	4.9	61	15.8
**• I get all the education about HBV infection and ways of protecting myself and my child through vaccination from the clinic**	246	63.9	51	13.2	39	10.1	49	12.7

### Summaries of descriptive statistics for the study variables

#### Levels of IMB constructs and HBV prevention practice

The findings displayed in Table 6 shows the summaries of the statistics computed for the study variables descriptively. It can be observed that the level of adequacy of information regarding the disease-specific knowledge measured on 12 point maximum scale of reference is average ($\overline{X }=$ 6.29, 95% CI = 5.97 ± 6.61), denoting 52.4% of the complete level of disease-specific knowledge expected from the respondents. The level of motivation ($\overline{X }=$ 17.71, 95% CI = 17.10 ± 18.31), behavioural skills ($\overline{X }=$ 12.88, 95% CI = 12.39 ± 13.37) and prevention practices ($\overline{X }=$ 15.62, 95% CI = 15.037 ± 16.20) were equally reported with a prevalence rate of 53.7%, 61.3% and 52.1% respectively (Refer to Table 6 for full details).

**Table 6:** Summaries of descriptive statistics computed for the respondents

Respondents in this study *N* = 385										
Variables	**Max. Score on Reference scale**		$\overline{{\varvec{x}} }$**(SE)**		** ± SD**	**Variance**		**95% confidence-interval**		**Prevalence**
Level of Information-Adequacy	12		6.29(0.163)		3.206	10.279		5.97–6.61		52.4%
Level of Motivation	33		17.71(0.308)		6.045	36.546		17.10–18.31		53.7%
Level of Behavioral Skills	21		12.88(0.250)		4.910	24.113		12.39–13.37		61.3%
Level of HBV infection prevention practices	30		15.62(0.298)		5.845	34.169		15.03–16.20		52.1%
Relationship between information-adequacy, motivation, behavioral skills and Prevention practices of HBV infection using Regression Analysis										
Variables		**B**		**R**^**2**^			**F-value**		***p*-value**	
Disease-specific Knowledge		0.574		.629			214.897		< .001*	
Motivation (Perception)		0.97							.014*	
Behavioral Skills		0.559							< .001*	

Note: *significant at *p* < 0.05

#### Relationship between IMB constructs and HBV infection prevention practices using linear regression analysis

##### Dynamics of HBV prevention practices using IMB constructs

**Fig. 2:**
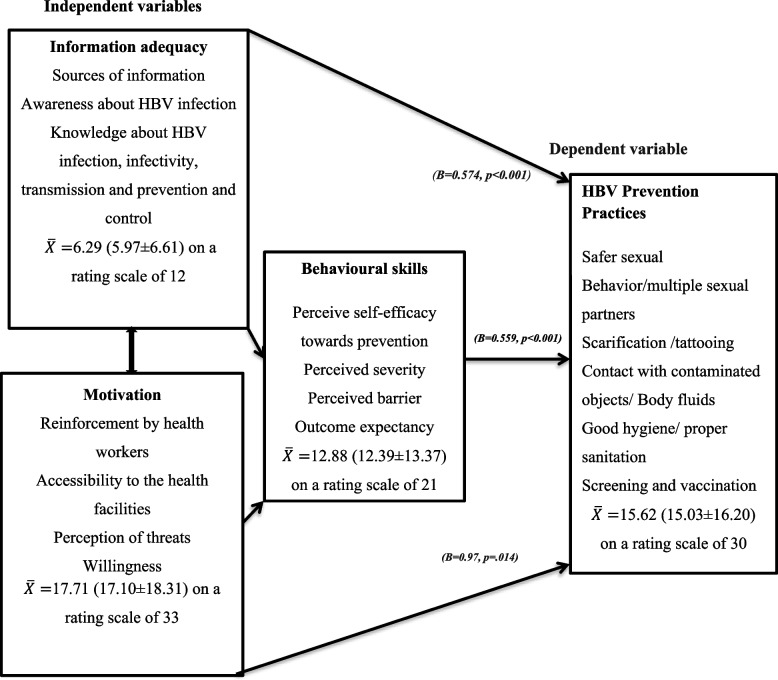
Conceptual framework derived from the information-motivation-behavioural skills model driving the study

Table 6 also provides the *R*^2^ value from the simple correlation which demonstrated that 62.9% of the total change in the prevention practices can be explained by the IMB-constructs. F-value of 214.897 with a significance level of (*p* < 0.001) from analysis of variance indicated that the regression model predicts prevention practices statistically and significantly well. Furthermore, the beta coefficient values for all predictors i.e. knowledge (B = 0.574; *p* < 0.001), perception (B = 0.97; *p* = 0.014) and behavioural skills (B = 0.559, *p* < 0.001) showed a statistically significant positive relationship with the prevention practices. Hence, they are covariant and the model guiding the study is obeyed (refer to Fig. 2).This denotes that the more each and every predictor increases, the more the prevention practice increase (see Table 6 for details).

Findings reported in Fig. 1 on vaccination uptake showed that only 13% of the respondents had vaccinated completely against HBV infection; less than 30 of the 385 (7.53%) respondents had vaccinated twice and only 5.19% of them reported to have taken the first dose of HBV vaccination. Generally, it is observed that an overwhelming majority of the respondents (74.03%) had never been vaccinated against HBV infection prior to this study.

The study applied this model of health psychological behaviour (IMB) to assess the awareness and HBV specific knowledge under the information adequacy. Perception, among others under motivation, the perceived self-efficacy, severity and outcome expectancy under the Behavioural skills as the independent variables to predict the prevention practices of HBV infection (i.e. the dependent variable) among the pregnant women (see Fig. 2 for details).

## Discussion

### Information-adequacy

The study sought to assess knowledge of the participants regarding HBV infection, their perceptions of consequences of an infection, and behavioural skills as predictors of HBV infection prevention practices considering the potential inherent in vertical transmission during pregnancy. This study captured data from three hundred and eighty-five (385) expectant mothers attending Lubaga Hospital for antenatal care and the findings revealed that majority of them demonstrated high awareness of HBV infection. Predominantly, they heard about the infection before pregnancy from places like hospitals, the community and from the public. Regarding the disease-specific knowledge, they demonstrated a fairly average knowledge level with a prevalence of 52.4% reported from a mean knowledge score of 6.29 (5.97 ± 6.61) as measured on a maximum score reference scale of 12. This fairly average knowledge level on hepatitis B infection is not in line with the unacceptable low level of knowledge displayed by pregnant women in a recently conducted study in Uganda by Nankya-Mutyoba et al., [15] and with others conducted within sub-Saharan Africa like that of Ali Abdulai and others [22]; Elsheik et al., [23]; Frambo et al., [24] and Dun-Dery et al., [25]. However, nearly similar to this average knowledge score is the report from the study conducted in Limbe Health District, Cameroon by Mbouamba-Yankam et al., [26]. In line with this finding, is the good knowledge level reported previously in a different geographical setting in Cameroon by Talla Paul et al., [27].

Due to the above-average level of knowledge of the respondents in this study, and in view of their awareness prior to this study, majority were still unable to identify some of the basic features of the mode of transmission including sexual transmission. Also, almost all the respondents did not know that fever could be an important sign of HBV infection and more than half of them did not also know that liver cancer risk is linked with being infected with hepatitis B virus infection. Knowledge gaps however, exist regarding reasons why at-risk population such as pregnant women and infants need to be protected from getting infected. Some (53%) of them in response felt that they can pass the infection to the baby through drinking water. Similarly, 3 out of every 4 pregnant women believed that it is because she may likely not be able to carry the pregnancy to term. Further, the role of vaccination in breaking the transmission of the infection vertically from mother to child as well as reducing the risk of chronicity could be hampered if the doses are not completed. Approximately half of the respondents did not know the number of doses required to complete the series of vaccination against hepatitis B thereby responding that just one or two doses are required whereas knowledge of vaccination has been previously reported to be a significant predictor of vaccination uptake [28]. This means that people who are adequately informed about vaccination are likely to uptake vaccine compared to those who are not knowledgeable about HBV vaccine. Health education in this setting still has a lot of gap to fill regarding the population of pregnant women’s knowledge of vertical transmission and the adequacy of the general awareness of HBV infection.

Information-adequacy about HBV infection in pregnant women population is a significant aspect of the strategies involved in micro-elimination of HBV towards elimination of the vertical transmission of HBV infection by 2030. The findings from this current study imply a control policy for hepatitis B in Uganda and similar regions within Sub-Saharan Africa where HBV infection education during antenatal visits, testing or vaccine uptake by pregnant women as well as administration of vaccination dose at birth for neonates are missing.

The average but inadequate knowledge about hepatitis B infection demonstrated by the respondents in this study is a missed chance to adequately inform expectant mothers on the consequences of hepatitis B infection complications on an expectant mother as well as the developing foetus. The issue might be due to the nature of the contacts expectant mothers have during antenatal clinic visits with the health facility which implies a need for health promotion intervention to target the observed knowledge deficiencies and improve the information-adequacy of the seriousness of HBV infection in order for the effective HBV infection prevention and control not to be hampered in Uganda. Similar studies by Ali Abdulai et al., [22] and Nankya-Mutyoba et al., [15] called for the same approach.

### Motivation (Perception)

Additionally, to the knowledge level, this study reported an averagely positive perception towards the prevention practices of hepatitis B infection among the respondents, displaying a prevalence of 53.7% ($\overline{X }=$ 17.71, 95% CI = 17.10 ± 18.31) on a rating scale of 33. This finding happens to be strongly concurring with the reports from previously conducted studies like that of France population by Brouard et al., [29], Nigeria by Chingle et al., [30] and Iran [31]. Contrary to the report of perception from this finding, is the low and incorrect perception towards HBV infection and liver carcinoma among similar population within central and northern Uganda reported in the study conducted by Nankya-Mutyoba et al., [32]. This present study’s results prove beneficial for interventions targeted at pregnant women’s perception.

Many of the respondents have a low perception towards the likelihood of contracting HBV infection and being predisposed to the risk of liver carcinoma, in spite of knowledge of the known link between liver carcinoma and hepatitis B. Despite the fact that 77% of the 385 study participants believe that vaccination of their baby at birth can offer protection against HBV infection and lower the risk of liver cancer, approximately half of them are motivated and willing to go for hepatitis B screening and vaccination simply due to recommendation by a health worker. Only a third of the respondents had a very high likelihood of taking their child for up taking HBV vaccine as a part of scheduled routine immunization for infant. This is a proportion a bit lesser than the 60.1% of respondents willing to do such in a report from the previous conducted study in obstetric population in Vietnam [33]. However, this study boasts of a less than 2% higher sample size than the 380 of Hang pham and others. This implies the need for prioritizing expectant mothers and mothers’ education in subsequent health campaigns with the aim of reducing wrong perceptions and improving the coverage of vaccine uptake at birth in Central Uganda.

The low likelihood of vaccinating themselves and their babies could be linked to their perception that vaccines circulating in Kampala are fake; as noted from some respondents. However, the 20% low proportion of the total respondents willing to get screened for HBV over the next 365 days is another issue reported in this study. It could be related to the misperception of the respondents that could be linked to their notion of the cost of screening and vaccination. Since they do not perceive themselves to being predisposed to hepatitis B infection or liver cancer risk, then their low likelihood of screening and vaccinating themselves might be justified. Hence, there is a need that the interventions directed towards HBV prevention focus on the perception of this population sub-group to initiate risk communication and strategies aimed at reducing this risk. It has been evidently shown in this study that there could be some other forms of motivation towards HBV prevention regardless of their low perception of risk which generally influenced the average score level of motivation recorded in this study. This has implications for health workers’ impacts and involvement toward shaping the perception of pregnant women and motivating them to seek testing services for HBV infection, uptake vaccine for their babies and initiate prevention practices of HBV infection.

### Behavioural skills

The findings regarding the level of behavioural skills towards HBV infection prevention practices among our respondents showed high behavioural skills with a prevalence rate of 63.1%. This is an indication that the respondents displayed good behavioural skills necessary for adopting a health-promoting behaviour. In terms of perceived severity, relatively half of them strongly agreed that hepatitis B is a serious infection that can be fatal. Also, should they develop cancer of liver, it will be critical more than any other complications that endangers career and deteriorate financial status. This report is almost in concord with the high perceived severity reported by Nankya-Mutyoba et al., [32] and a more higher perceived severity reported in the study conducted by Balegha et al., [34], although the discrepancy in the findings might be as a result of the differences in the population of study since Balegha et al., [34] focused their study on nursing students in contrast to pregnant women as employed in this study.

Regarding the outcome expectancy, roughly half of the respondents agreed that they were confident that getting screened for hepatitis B infection will help them discover and treat the infection quickly before it leads to liver carcinoma. Despite the all-inclusive high behavioural skills score level, just one-third of the respondents strongly agreed that they confidently believed that vaccinating their child against HBV infection will get them no worries about the baby developing carcinoma of the liver during adulthood. This report may be as a result of the gaps in their knowledge about liver cancer and the relationship with HBV infection as well as the efficiency of vaccination in preventing the infection. While a third of the total respondents did not perceive not experiencing liver infection symptoms as a barrier to testing themselves for hepatitis B and going for vaccination, 4 out of every 10 pregnant women strongly believed that miscarriage will result from getting vaccinated for HBV during pregnancy. This could be linked to the fact that some are of the perception that available vaccines in Kampala are fake and they do not want to put themselves and their unborn babies at risk. This implies that hepatitis B prevention education policy should direct communication on effective vaccination and information on location and availability of vaccination services to the public and the vulnerable group in order to improve the uptake of vaccination among the obstetric populations.

Furthermore, an overwhelming majority of the respondents displayed an encouraging self-efficacy by strongly agreeing to the item inquiring about their certainty of taking their child for all the required vaccinations regardless of the distance of the immunization centre from their places of residence. This is due to the fact that most of them who seem to be adequately informed about HBV infection rejected their high likelihood of not going for vaccination because the clinic is far from where they live and this knowledge could have reformed their perception giving them the confidence in preventing themselves and their unborn babies come what may. Simultaneously, most of them (due to the mentioned fact) strongly agreed that they were certain of going for HBV screening irrespective of the payment the screening required, although the perceived self-efficacy is not as high compared to the one reported by Nankya-Mutyoba et al., [32]. However, it has together with their high outcome expectancy, low perceived barrier and high severity influenced the total high score of 63.1% generated for their behavioural skills. This implies that if the benefit of vaccination is clearly perceived by people the same way they perceive the severity of the infection if they are not protected and without having any hindrance towards prevention, they are likely going to initiate prevention practices. Attention of HBV prevention education policy should however be paid to communicating the complications of HBV infection, the need and benefit of vaccination to the pregnant women as this will influence their self-efficacy and in turn have impact in their uptake of HBV prevention services. Therefore, awareness of the pregnant women must be raised and necessary HBV vaccine-related important information must be provided to them as a significant approach in order for vaccination to be scaled up among this high-risk population group.

### Hepatitis B virus infection prevention practices

Reported findings from this study show that the respondents in the study demonstrated an average score level for prevention practices towards HBV infection with a prevalence of 52.1% ($\overline{X }$=15.62, 95% CI = 15.03 ± 16.20) when measured on a 30-points rating scale. This result contradicts poor practices that numerous studies have previously reported within Sub-Saharan Africa like that of Elsheik et al., [23]; Mbouamba-Yankam et al., [26] and Balegha et al., [34]. Almost in line with this finding, is the relatively better or safe prevention practices reported by Talla Paul et al., [27] and Mursy and Mohamed [35]. However, the latter [35] focused on nurses and midwives in contrast to expectant mothers employed in our study. The general average level of prevention practices reported in this study leaves no much concern. However, the frequency of some of the respondents hepatitis B prevention practices do leave a lot to worry about considering the fact that less than 4 out of every 10 respondents avoid unprotected sex with the use of barrier protective measure very often which might be due to their religious belief. This is based on the fact that majority of the respondents were reported to be Catholics followed by Muslims and the idea of using such protective wears might not be too welcoming. However, it will contribute less to their risks of being infected since majority of them reported to practice safer sex often and avoid having more than one sexual partner.

This finding implies that the national prevention policy may alternatively intensify approaches regarding the prevention, complications, and risk factors by reaching out to individuals within the grassroots. Suggested places include sensitization in worship centres through leaders of religious groups. This would enhance the continuous dissemination of information through person-focused approach regarding the prevention and control of hepatitis B infection.

While 7 out of every 10 respondents avoid having tattoos on their body just around one-third of the respondents do not always share and reuse needle. The poor practice of reusing needles is a reflection of their lack of knowledge concerning the predisposing risk factors of HBV infection. Amazingly, half of them claimed to avoid casual contact with HBV infected person very often and 42.6% of the respondents often avoided sharing food with infected person in order to prevent the infection. Both practices however, are not associated with the transmission of the infection and they do not know because their knowledge of the transmission of HBV is not adequate. Regarding routine screening for HBV detection, more than half of the respondents do not undergo regular routine screening. Similarly, nearly three-quarter of the total respondents do not often complete recommended HBV infection vaccinations for their children as part of scheduled routine immunization required for infants. This self-reported practice might be due to their perception towards vaccination. Also, just 12.7% of the 385 pregnant women claimed to have derived information from the clinic about HBV infection as well as ways of protecting themselves and their children. This is indeed, a gap that is needed to be filled at the health facility for majority showed from their perceptions that they can be motivated to initiate prevention practices like screening and vaccination should they be recommended by a health worker. This implies a need for more personalized HBV health education by the health workers who have been directed to cater for expectant mothers during antenatal visits in order to adequately inform them about the infection dynamics and how to prevent themselves and their unborn babies. Evidently from our present study, it was observed that the overwhelming majority of the expectant mothers had not been vaccinated for HBV prior to the study denoting a very low uptake of HBV vaccination. This low uptake of vaccination is comparable to the report from a study previously conducted by [36] in Northern Uganda. This suggests the need for public health interventions informing pregnant women adequately on HBV education as well vaccination.

Findings reported on the study variables relationship demonstrated a statistical significant predicting-association between the disease-specific knowledge, motivation, behavioural skills and HBV infection prevention practices among the study respondents. Numerous previously conducted studies have reported significant relationships between knowledge of HBV as well as prevention practices, which is in agreement with this study like that of [26, 37], while a study by [27] reported a non-significant relationship between knowledge and practice. Regarding perception predicting prevention practices, Chingle et al., [30] reported such in a study conducted in West Africa. However, the study only measured perception of risks unlike this present study that had some other constructs of motivation such as reinforcement by health worker among others, measured with perception. In addition to behavioural intentions that included self-efficacy, one of the major constructs in the behavioural skills measured in this study, perception was reported to be associated significantly with it in the studies conducted in Iran [31] and that of [32] conducted in Uganda. The all-inclusive significant predicting relationship observed in our study obeys the theoretical model that guided this study [13] which postulates that information-adequacy, motivation and behavioural skills are the leading predictors of health behaviours’ performance as well as adoption. Similarly, it states that when people become adequately informed; motivated towards performing an action and possess the necessary skills shaping behaviours, an enabling environment will be created vis-à-vis adopting or initiating practices that would positively promote their health. Contrarily, when individuals demonstrate poor information on health problems and performing action, they do not tend to be motivated and are also short of the required skills for shaping behaviours to perform effectively. Consequently, they will end up adopting and engaging in health risk practices that could bring about negative health outcomes. These findings have significant implications for HBV prevention policy and will contribute to filling a gap already known regarding usable facts like the inadequate knowledge and misperceptions including the focused areas of intervention that will help to inform programs aimed at reinforcing HBV infection prevention behaviours among pregnant women.

This study will be the first of its kind hitherto as there are no studies in Uganda and across literature search that has reported to use this theoretical model (IMB) combining the explored variables to predict HBV infection prevention practices displayed by pregnant women.

### Lessons learned from the study

Globally, human infection transmissions are effectively prevented when adequately vaccinated with the most appropriate vaccines, especially, among pregnant women and infants who are most vulnerable. The concerns about HBV infection is a global threat among at-risk populations and the lesson learned in this study has implications throughout the African continent, in the first instance, where the psycho-cognitive dispositions and behavioural responses are like the population studied in Uganda at the health facility. No doubt, a multi-country study may need to be conducted or, at best, a systematic review and meta-analysis of the subject of consideration in the study to ascertain the true profile of HBV vaccine coverage and antecedent factors associated with identified outcomes.

## Conclusion

This study reported an average but inadequate level of hepatitis-B-specific knowledge, a general positive perception with some negative ones and good skills for adopting right behaviours to avoid HBV infection which in turn, informed averagely acceptable prevention practices among the respondents in the study.

## Recommendation

The gaps in the knowledge and the incorrect perceptions towards HBV infection prevention practices among the respondents in this study necessitate the need for personalized health education during antenatal visits including regular public health campaigns. This will adequately inform the expectant mothers about hepatitis B infection, motivate them to act towards health-promoting behaviours as well as equip them with the necessary and effective behavioural skills that will translate to positive health outcomes for them towards reducing the vertical transmission of this infection.

## Supplementary Information

**Additional file 1.** 

## Data Availability

The quantitative datasets analysed for this current study are available from the corresponding author upon reasonable request.

## Abbreviations

ANC: Antenatal Care
GBD: Global Burden of Diseases
HBsAg: Hepatitis-B-surface Antigen
HBV: Hepatitis B virus
HIV: Human-Immuno-Deficiency Virus
IMB: Information-Motivation-Behavioural Skills Model
LHREC: Lubaga Hospital Research Ethical Committee
WHO: World Health Organization
