# Supplementary material for: Dynamics of Hepatitis B infection prevention practices among pregnant women attending antenatal care at Lubaga Hospital Kampala, Uganda using the constructs of information-motivation-behavioural skills model
Source: BMC Public Health. 2022 Dec 1;22:2243. doi: 10.1186/s12889-022-14723-3 (PMC9714095; doi:10.1186/s12889-022-14723-3)
Supplement: Supplementary file 1 — Additional file 1. [file 12889_2022_14723_MOESM1_ESM.pdf]

# LUBAGA HOSPITAL

Trustees of the Archdiocese of Kampala

31 August 2020

**REF: LHREC/2020/ 07**

Mr. Afolabi Ismail Bamidele
Cavendish University
Kabalagala, Kansanga

Dear Afolabi,

**RE: LHREC PROTOCOL 2020/07 DISEASE SPECIFIC KNOWLEDGE, PERCEPTION AND BEHAVOURIAL SKILLS AS PREDICTORS OF HEPATITIS B PREVENTION PRACTICE AMONG PREGNANT WOMEN ATTENDING ANTENATAL CARE AT LUBAGA HOSPITAL, KAMPALA, UGANDA**

This is to inform you that the Lubaga Hospital Research Ethics Committee (LHREC) has approved the above research study. The approval period is **from 24th August 2020 to 23 August 2021 is LHREC/2020/04.Please be sure to reference either this number in any correspondence with the LHREC.**

Continued approval is conditional upon your compliance with the following requirements:

1) A copy of the **Informed Consent Document**, approved as of 24 August **2020** is enclosed. No other consent form should be used. It must be signed by each subject prior to initiation of any protocol procedures. In addition, each subject must be given a copy of the signed consent form.

2) All protocol amendments and changes to approved research must be submitted to the LHREC and not be implemented until approved by the LHREC except where necessary to eliminate apparent immediate hazards to the study subjects.

3) Significant changes to the study site and significant deviations from the research protocol and all unanticipated problems that may involve risks or affect the safety or welfare of subjects or others, or that may affect the integrity of the research must be promptly reported to the LHREC.

Please complete and submit reports to the LHREC as follows:

a) Renewal of the study - completes and returns the Continuing Review Report-Renewal Request (Form 404A) at least 8 weeks prior to the expiration of the approval period. The study cannot continue after **23/08/2021** until re-approved by the LHREC.

b) Completion, termination, or if not renewing the project- send the report upon completion of the study.

Please call me if you have any questions about the terms of this approval.

Yours sincerely,

Dr. Michael Okello
**MEDICAL DIRECTOR**
**For: LHREC CHAIRMAN**

LUBAGA HOSPITAL RESEARCH
ETHICS COMMITTEE
CHAIRMAN
P.O. Box 14130 Kampala
Tel: 0204 234 800
Date……………………
Sign……………………

P.O. Box 14130, Kampala-Ug.
0200244800
info@lubagahospital.org
www.lubagahospital.org
Toll Free: 0800 388 888

*Service with love*
